# Gastroesophageal Junction Adenocarcinoma Presenting With De Novo Metastatic Disease and Ovarian Metastasis in a Young Woman With Atypical Chest Pain

**DOI:** 10.7759/cureus.109095

**Published:** 2026-05-18

**Authors:** Yanina Nikolaus, Mustafah Qureshi

**Affiliations:** 1 Pathology, Marshall University Joan C. Edwards School of Medicine, Huntington, USA

**Keywords:** early-onset cancer, esophageal adenocarcinoma, gastroesophageal junction adenocarcinoma, krukenberg tumor, metastatic gastroesophageal cancer, molecular profiling, ovarian metastasis, pd-l1, pembrolizumab

## Abstract

A 33-year-old woman presented with progressive dysphagia and pleuritic chest pain, initially undergoing an unrevealing cardiopulmonary evaluation. Persistent symptoms prompted upper endoscopy, which demonstrated an ulcerated gastroesophageal junction lesion, with biopsy confirming invasive moderately to poorly differentiated adenocarcinoma. Immunohistochemical studies showed intact mismatch repair protein expression and negative human epidermal growth factor receptor 2 and Claudin 18.2, while programmed death-ligand 1 expression was markedly elevated (tumor proportion score 80%; combined positive score 80). Comprehensive next-generation sequencing revealed no actionable genomic alterations. Staging positron emission tomography demonstrated widespread metastatic disease, including involvement of the regional lymph nodes, liver, and ovary. The patient was treated with combination chemotherapy and immune checkpoint inhibition. This case highlights an atypical presentation of advanced gastroesophageal junction adenocarcinoma in a young adult and underscores the importance of early endoscopic evaluation in patients with persistent dysphagia, as well as recognition of uncommon metastatic patterns and the therapeutic implications of biomarker profiling.

## Introduction

Gastroesophageal junction adenocarcinoma predominantly affects older men and is commonly associated with gastroesophageal reflux disease and Barrett's esophagus. Despite advances in diagnosis and treatment, the global burden of esophageal and gastroesophageal junction malignancies remains substantial, with increasing incidence reported over recent decades [1-3]. Although these tumors are typically diagnosed in older adults, increasing recognition of early-onset gastrointestinal malignancies has been described, and younger patients may present with more advanced disease at diagnosis [4].

Ovarian metastases are most frequently associated with gastric adenocarcinoma and are less commonly reported in association with gastroesophageal junction primaries [5,6]. Comprehensive molecular profiling now plays an increasingly important role in the management of advanced gastroesophageal malignancies, including the assessment of mismatch repair status, human epidermal growth factor receptor 2 expression, and programmed death-ligand 1 expression, which may influence therapeutic decision-making [7].

## Case presentation

A 33-year-old woman presented with a one-month history of progressive dysphagia to solid foods accompanied by pleuritic chest pain. Initial computed tomography angiography excluded pulmonary embolism and did not demonstrate acute cardiopulmonary abnormalities (Figure 1). Retrospective review did not identify convincing radiographic findings suggestive of gastroesophageal junction malignancy on the initial study. Given persistent symptoms, upper endoscopy was performed and revealed an ulcerated lesion at the gastroesophageal junction (Figure 2). Biopsy demonstrated invasive moderately to poorly differentiated adenocarcinoma. Family history was notable for colon cancer in a first-degree relative.

**Figure 1:**
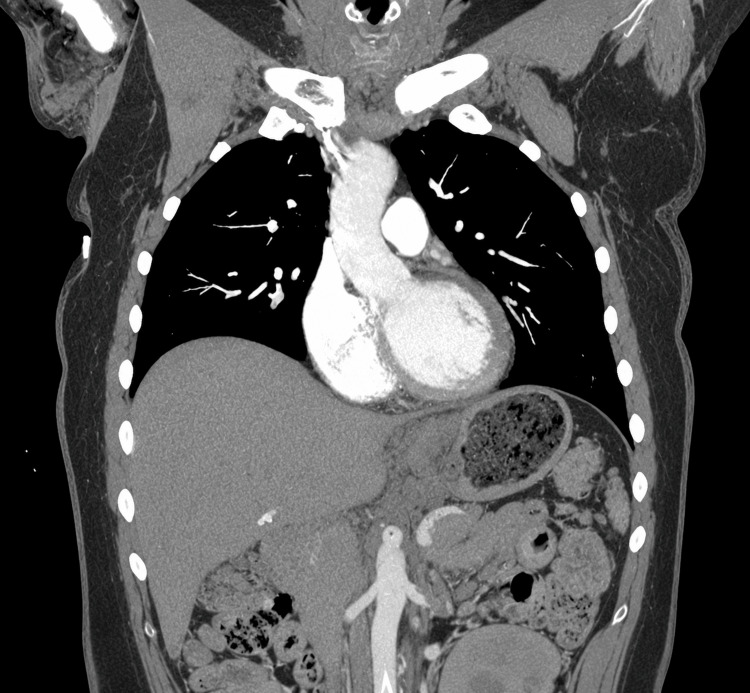
Initial computed tomography angiography demonstrating no acute cardiopulmonary abnormality at presentation

**Figure 2:**
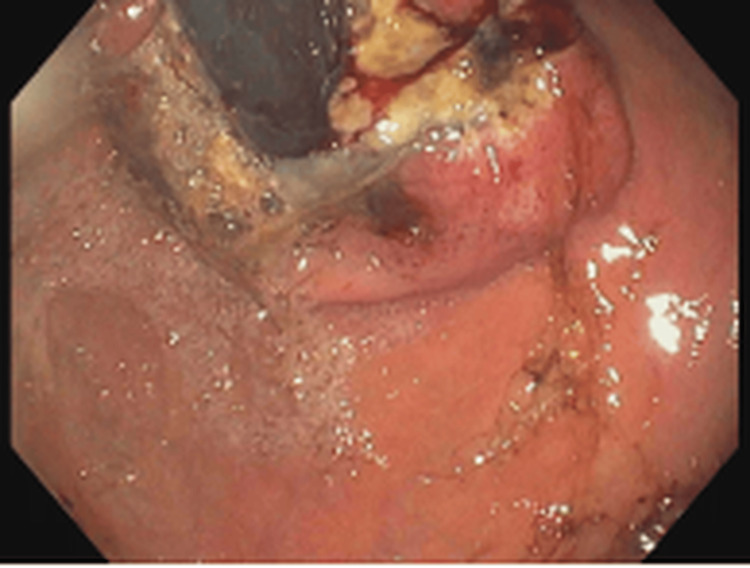
Endoscopic image demonstrating an ulcerated, friable mass at the gastroesophageal junction causing luminal narrowing

Investigations

Histologic examination demonstrated infiltrative malignant glandular structures with a desmoplastic stromal response (Figure 3). Higher magnification showed marked cytologic atypia with enlarged hyperchromatic nuclei, irregular nuclear contours, and glandular formation (Figure 4).

**Figure 3:**
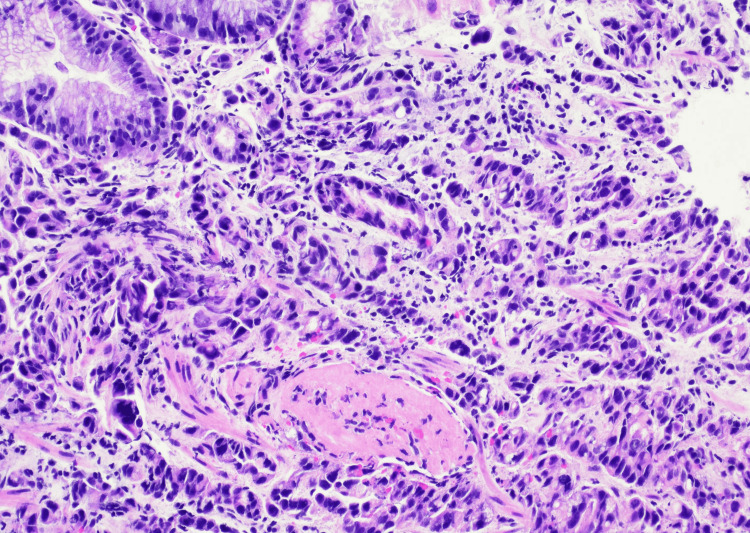
Histologic section of the gastroesophageal junction lesion showing infiltrative malignant glandular structures embedded within a desmoplastic stroma (hematoxylin and eosin stain, ×200 objective)

**Figure 4:**
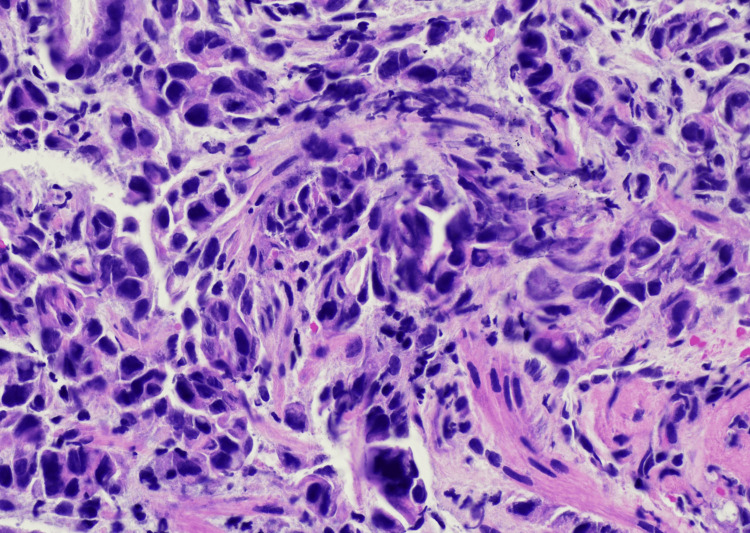
Higher-power view demonstrating cytologic atypia with enlarged hyperchromatic nuclei, irregular nuclear contours, and glandular formation consistent with moderately to poorly differentiated adenocarcinoma (hematoxylin and eosin stain, ×400 objective)

Immunohistochemical studies showed intact nuclear expression of mismatch repair proteins. Human epidermal growth factor receptor 2 and Claudin 18.2 were negative. Programmed death-ligand 1 immunohistochemistry performed using the DAKO PD-L1 22C3 pharmDx assay demonstrated strong membranous staining (tumor proportion score 80%; combined positive score 80). Combined positive score was used to guide therapeutic decision-making regarding the incorporation of immune checkpoint inhibitor therapy. Comprehensive next-generation sequencing did not identify clinically actionable genomic alterations (Table 1).

**Table 1:** Biomarker and molecular profile of the gastroesophageal junction adenocarcinoma

Test/marker	Result	Interpretation
Mismatch repair protein immunohistochemistry	Intact nuclear expression	Mismatch repair proficient
Microsatellite status	Microsatellite stable	No evidence of microsatellite instability
Human epidermal growth factor receptor 2 immunohistochemistry	Negative; score 0	No human epidermal growth factor receptor 2 overexpression
Claudin 18.2 immunohistochemistry	Negative; score 0%	No Claudin 18.2 expression
Programmed death-ligand 1 immunohistochemistry	Tumor proportion score 80%; combined positive score 80	High programmed death-ligand 1 expression
Tumor mutational burden	5.8 mutations/megabase	Low-intermediate tumor mutational burden
Next-generation sequencing	MET copy number gain; TP53 splice-site variant; LRP1B truncating variant; CCND1, FGF3, and FGF4 copy number gains	No clinically actionable genomic alteration was identified in this clinical context

Staging positron emission tomography demonstrated marked hypermetabolic activity involving the distal esophagus and gastroesophageal junction with metastatic involvement of the regional lymph nodes, liver, and ovary, consistent with disseminated disease (Figure 5).

**Figure 5:**
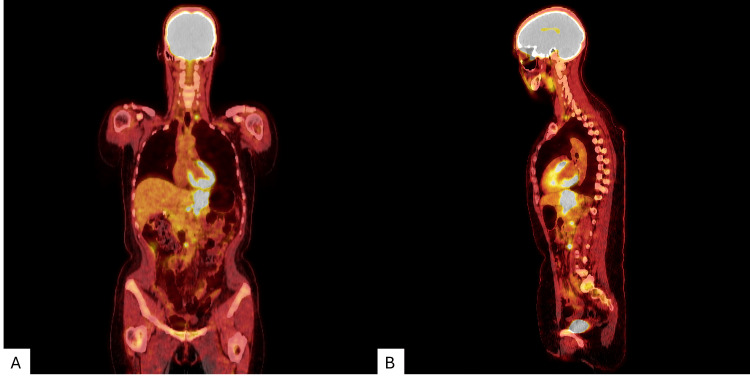
Positron emission tomography/computed tomography demonstrating hypermetabolic gastroesophageal junction adenocarcinoma with metastatic disease Fluorodeoxyglucose positron emission tomography/computed tomography demonstrating marked hypermetabolic activity involving the distal esophagus and gastroesophageal junction with associated metastatic adenopathy and distant metastatic disease. Coronal (A) and sagittal (B) fused images are shown.

Treatment

The patient was initiated on modified fluorouracil, leucovorin, and oxaliplatin in combination with pembrolizumab administered every two weeks, in accordance with contemporary first-line treatment strategies supported by phase 3 trials demonstrating improved outcomes with immune checkpoint inhibitors. An esophageal stent was placed for palliation of obstructive symptoms. Supportive care included antiemetics and long-acting opioid therapy for cancer-related pain.

Outcome and follow-up

At the most recent follow-up, the patient remained on systemic therapy with modified fluorouracil, leucovorin, and oxaliplatin in combination with pembrolizumab. She reported stable swallowing function with continued ability to tolerate oral intake, and cancer-related pain was well controlled. Treatment was overall well tolerated, and her Eastern Cooperative Oncology Group performance status remained 1 [8].

Follow-up computed tomography obtained after systemic therapy demonstrated persistent distal esophageal thickening with an interval decrease in previously identified metastatic lymphadenopathy (Figure 6). A previously noted hepatic lesion was not clearly visualized on the most recent study. The primary tumor at the gastroesophageal junction remained difficult to assess due to the presence of an esophageal stent.

**Figure 6:**
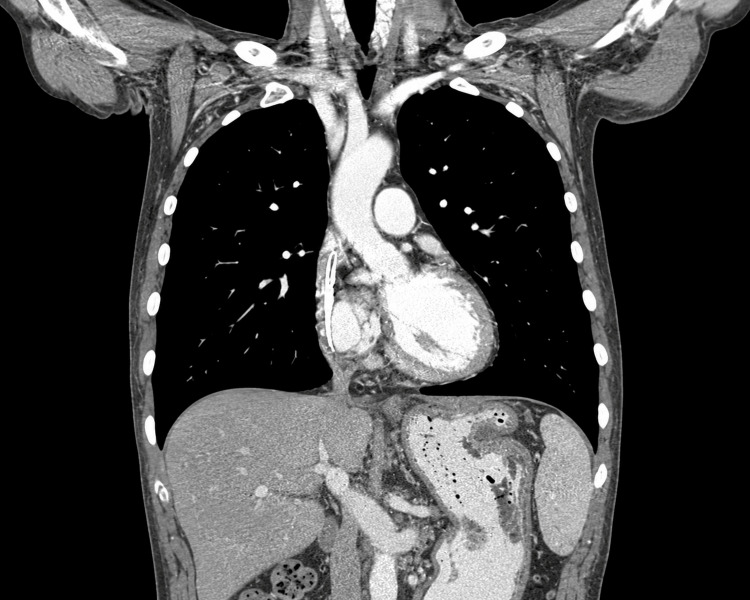
Follow-up computed tomography obtained after systemic therapy

The patient continues on systemic therapy, with plans for repeat positron emission tomography imaging in six weeks. Given the observed response, referral to radiation oncology has been initiated for consideration of consolidative radiation therapy to sites of residual disease, followed by continuation of immunotherapy.

## Discussion

Esophageal and gastroesophageal junction adenocarcinomas are most commonly diagnosed in older men, with rising global incidence documented over recent decades [1-3]. However, early-onset gastrointestinal malignancies are increasingly recognized, and younger patients are more likely to present with advanced-stage disease at diagnosis [4].

In this case, initial cardiopulmonary imaging performed for pleuritic chest pain was unrevealing, and the diagnosis was established only after persistent dysphagia prompted endoscopic evaluation. This highlights the importance of maintaining clinical suspicion and pursuing early endoscopic evaluation in patients with persistent structural esophageal symptoms, regardless of age.

Ovarian metastases are most frequently associated with gastric adenocarcinoma and are less commonly reported from other upper gastrointestinal primaries [5,6]. Recognition of this metastatic pattern is important, particularly in younger patients presenting with adnexal lesions, as it may lead to diagnostic confusion with primary ovarian neoplasms.

Molecular profiling is essential in the contemporary management of advanced gastroesophageal junction adenocarcinoma [7]. The tumor in this case demonstrated intact mismatch repair protein expression and lacked human epidermal growth factor receptor 2 expression, limiting targeted therapeutic options. However, programmed death-ligand 1 expression was markedly elevated. High programmed death-ligand 1 expression has been associated with improved response to immune checkpoint inhibitors, supporting the incorporation of immunotherapy into first-line treatment strategies in advanced disease [9-11].

Learning points

This case highlights that gastroesophageal junction adenocarcinoma can occur in younger adults and may initially present with disseminated metastatic disease. Persistent dysphagia warrants prompt endoscopic evaluation regardless of patient age. Ovarian metastasis should be considered in patients with upper gastrointestinal malignancies presenting with adnexal lesions. Comprehensive molecular profiling remains essential in advanced disease to guide biomarker-driven therapeutic strategies, particularly when markedly elevated programmed death-ligand 1 expression may influence first-line treatment selection, including the use of immune checkpoint inhibitors.

## Conclusions

This case highlights de novo stage IV gastroesophageal junction adenocarcinoma presenting in a young adult with markedly elevated programmed death-ligand 1 expression and ovarian metastatic involvement. This case also underscores the importance of maintaining clinical suspicion in younger patients presenting with persistent esophageal symptoms. Comprehensive molecular profiling remains essential in advanced gastroesophageal malignancies, particularly when actionable therapeutic biomarkers may influence first-line systemic treatment strategies.
